# Liquid-Infused Microgrooved Slippery Surface Ablated by One-Step Laser Irradiation for Underwater Bubble Directional Manipulation and Anisotropic Spreading

**DOI:** 10.3390/mi12050555

**Published:** 2021-05-13

**Authors:** Wei Liu, Xuehui Chen, Yunlong Jiao

**Affiliations:** 1 College of Mechanical and Electrical Engineering, Anhui Jianzhu University, Hefei 230601, China; wliu@hfcas.ac.cn; 2 Institute of Tribology, Hefei University of Technology, Hefei 230009, China

**Keywords:** liquid-infused anisotropic slippery surface, one-step laser irradiation, underwater bubble manipulation, anisotropic spreading

## Abstract

A pitcher plant is a kind of liquid-infused porous surface that imparts an excellent directional manipulation ability to in-air droplets or underwater bubbles, so it has attracted researchers’ attention in both academic and industrial issues. In this work, a kind of liquid-infused anisotropic microgrooved slippery surface (LIAMSS) was fabricated through one-step femtosecond laser irradiation and lubricant coating technology. On the inclined LIAMSS, the underwater bubbles show great directional motion and anisotropic spreading ability under the effect of buoyancy. It should be noted that the interaction between the air and the lubricant layer plays a dominant role in determining the attachment and the movement of the underwater bubble, which could be ascribed to the competition between the adhesion resistance induced by contact angle hysteresis and the drive force induced by buoyancy. Additionally, the bubble shows obvious anisotropy on the LIAMSS with the increase in volume because of the restriction of the slippery area, and the bubble contact angle perpendicular to the grooved region is about 88^○^ when the bubble volume is 5 μL. We believe that the present findings would accelerate the application of this kind of bubble slippery surface in underwater gas collection and tail gas treatment.

## 1. Introduction

Underwater bubbles are significant liquid systems involved in solving both scientific and industrial issues [1,2,3,4,5], and play critical roles in the fields of wastewater treatment [6], elevating heat transfer in the ocean [7], catalytic reactions [8] and useful gas manipulation [9]. It is worth noting that the directional transportation of bubbles in a liquid environment is the key to achieving these multi-functionalities. Herein, inspired by natural organisms, functional surfaces with extreme wettabilities have been developed as a useful tool for controlling the movement of small bubbles [10,11,12,13,14,15,16]. For example, Pei et al. prepared an underwater unidirectional penetration film for gas separation by modifying the copper meshes, which is defined as the “bubble diode” [10]. Zhang et al. designed a kind of tapered slippery surface induced by two biomimetic principles (cactus and Picher plant) to realize continuous underwater bubble transport [11]. Recently, Chen et al. reported and fabricated a light-responsive slippery liquid-infused porous surface inspired by the pitcher plant to actuate the movement of bubbles in any direction, the mechanism of which relies on a giant wettability gradient force induced by temperature difference [12]. In general, natural organisms offer diverse multiscale micro/nanostructures for humans to emulate, meaning the manipulation of bubbles in water can be realized.

Despite tremendous developments in the field of underwater bubble manipulation, it should be noted that the multiscale structure is recognized as the key element in determining the reproduction of these unique functions [17,18,19,20]. For instance, the lotus leaf has inspired a method to fabricate a hierarchical micro/nanostructure with super hydrophobicity, which shows a high affinity for air in water, so it could be used for capturing and collecting gas [17]. The pitcher plant has inspired the design of a liquid-infused slippery surface for underwater bubble unidirectional transportation [18], and the cactus has provided a needle-like structure to realize the self-transport of underwater bubbles induced by the Laplace pressure effect. However, these unique micro/nanostructures have brought about great challenges for micro/nanoprocessing methods, including lithography [21], electro-chemical deposition [22], plasma processing [23], etc. [24], which usually consist of multistep technology processes and can easily address environmental problems. The emergence of femtosecond laser ablation technology (FLAT) has brought a new processing strategy to the field of micro/nanomanufacturing. It is worth noting that the femtosecond laser is a facile strategy for fabricating microstructures on the surfaces of various materials (e.g., metal [25], polymer [26], etc.). It can precisely control the size of microstructures by adjusting some crucial parameters, including laser power, scanning speed and scanning period. In addition, compared to the traditional chemical hydrophobized method, laser ablation is more competent for modifying surface wettability upon induction of multiple micro/nanostructures [27,28,29,30]. That is, a super-wettable surface realized by laser is made possible by the intrinsic properties of the material itself, while those realized by traditional chemical modifications are dependent on fluorine-doped solvents, thus the former is more stable than the latter. Through advanced micro/nanomanufacturing technology, we can replicate unique biomimetic micro/nanostructures to achieve diverse functional applications.

Herein, combined with FLAT and liquid-infused lubrication technology, we fabricate a kind of liquid-infused anisotropic microgrooved slippery surface (LIAMSS) to achieve the precise manipulation of underwater bubbles in a restricted region. Compared with superhydrophobic/superhydrophilic surfaces, SLIPS are very repellent to various liquids with different surface tensions [31,32,33], and thus serve as efficient aerophobic interfacial materials for bubble manipulation. It is worth noting that the mechanism of bubble directional transmission is mainly related to the competition between the driven force induced by the underwater buoyancy and the resistance force induced by the contact angle hysteresis. Due to the influence of bubble volume, the underwater buoyancy of the bubbles is different, so the bubble starts to move when the rotating angle of the slippery surface increases to the critical sliding angle. The smaller the bubble volume, the higher the critical sliding angle. Additionally, we have compared the water contact angles on the LIAMSS during the fabrication process, and the wetting states between the droplet and the surface are obviously different due to the existence of lubricant, which contributes to the variation in the contact angles on the slippery surface. Finally, we make use of a kind of microgrooved region on the LIAMSS to realize the anisotropic spreading of the underwater bubbles. It can be seen that the anisotropy of the bubble becomes more and more obvious with the increase in bubble volume, and the bubble contact angle perpendicular to the grooved region is about 88° when the bubble volume is 5 μL.

## 2. Materials, Methods and Fabrication Process

Materials: The fluorinert lubricant (FC-3283) and superhydrophobic coating materials (Glaco) were purchased from Xin Hua Tech. Co., Ltd. and they were used for the preparation of LIAMSS. The sample was a 6061 alloy sheet and it was cleaned in alcohol 15 min before the laser fabrication. The distilled water served as the contact angle measurement material.

Femtosecond laser manufacturing: The multiscale microgrooved structures on the metal surface were realized by line-by-line femtosecond laser direct writing, and the laser beam was produced by a regenerative amplified Ti:sapphire femtosecond laser system (Legend Elite-1K-HE, Coherent, CA, USA). It should be noticed that the height and the interval of the structures could be precisely controlled by adjusting the laser power, scanning spacing and speed. In general, the fast scanning speed and large scanning period could increase the fabrication efficiency. However, the multiscale micro/nanostructures are more easily induced at lower scanning speeds in shorter scanning periods, which is important for achieving extreme wettability on the microstructured surface. Therefore, in order to keep a balance between the fabrication efficiency and equality, the fabrication parameters were set at 350 mW, 100 μm and 2 mm/s.

**Figure 1:**
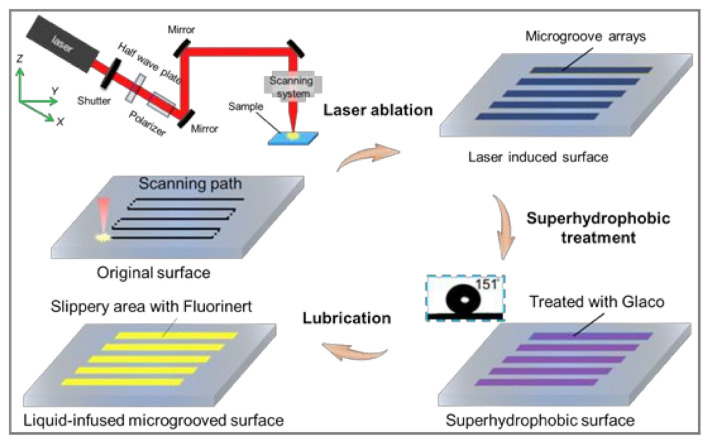
The fabrication process of liquid-infused anisotropic microgrooved slippery surface (LIAMSS), which mainly contains three steps: femtosecond laser ablation, superhydrophobic treatment and lubrication process. It should be noted that the superhydrophobic treatment is meant to reduce the surface energy of the LIAMSS such that the lubricant layer could remain stable in water.

Fabrication process of bionic liquid-infused slippery surface: The detailed fabrication process of the LIAMSS, derived from the design inspiration of the pitcher plant, is shown in Figure 1. It can be clearly seen that the process could be divided into three main steps: laser irradiation, superhydrophobic treatment and lubrication process. Firstly, the laser directly ablated the metal surface by line-by-line scanning and the multiscale micro/naostructures were formed on the original sample. Then, a kind of superhydrophobic reagent was evenly sprayed on the laser-induced sample to reduce surface energy and the sample showed superhydrophobicity with a contact angle of 151°. Thereafter, the lubricant (FC-3283) was dripped onto the superhydrophobic surface to form a thin oil layer on the surface, which is the key to fabricating the slippery surface. It is worth noting that the lubricant layer is stable on the surface as the result of the low surface energy in water. Due to the low surface energy, the water could not sink into the microstructured surface, and this would reduce the deterioration of lubricant. In short, to obtain a stable liquid-infused microstructured slippery surface, there are three criteria that must be satisfied [16,34]. First, the lubricant liquid must wet and stably adhere to the substrate. Second, the substrate must prefer the lubricant liquid over water. Third, the lubricant and test liquid must be immiscible.

Characterization: The microgrooved arrays on the alloy materials fabricated by one-step ablation were characterized by using an optical microscope and a field-emission scanning electron microscope (JSM-6700F, Japan). The contact angles of the water droplet and the underwater gas bubbles were measured by a contact angle meter under 50% humidity and 20 °C temperature, respectively. The droplet and bubble volumes were 5 μL. Additionally, in order to decrease the measuring error, the final results were defined by the average value of five measurements.

Directional transmission of underwater bubbles on the LIAMSS: The as-prepared samples were put underwater in a rectangular transparent box. An injector was employed to add bubbles into the LIAMSS. Through controlling the inclined angle of the samples, the bubbles could be made to slip along the restricted slippery surface under the effect of buoyancy. It should be noted that the detailed movement process was recorded by a digital camera from the side view.

## 3. Results

### 3.1. Surface Topography of the Laser-Ablated Sample

**Figure 2:**
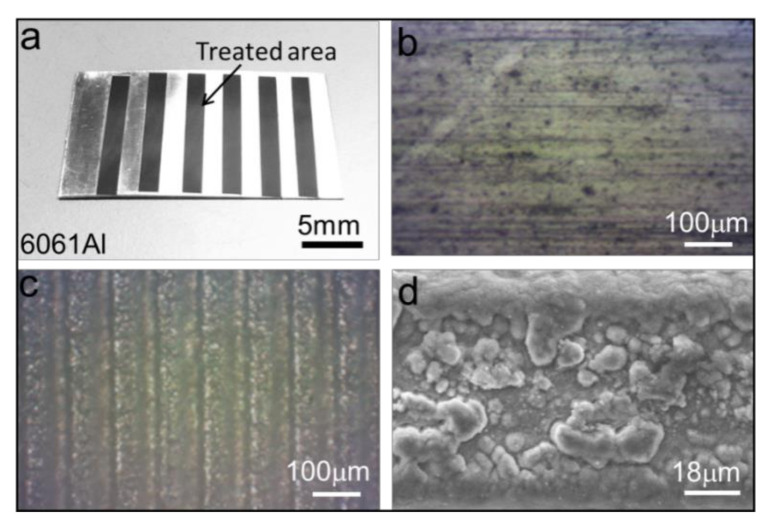
Surface topography of laser-ablated sample. (**a**–**c**) Optical image of original sample and laser-treated area on the 6061 alloy sheet. It can be seen that the microgroove arrays are regularly arranged in the treated area with a spacing of ~100 μm. (**d**) SEMs of laser-induced microgrooves, it can be seen that there are some nanoparticles (200~500 nm) induced by debris deposition on the microgrooves.

In order to fully display the surface topography of the laser-induced sample, the optical images and SEMs with different magnification were obtained by using a field-emission scanning electron microscope (Figure 2). It can be seen that the laser-treated area has obvious color variation compared with the untreated area after the ablation process (Figure 2a), and the microgroove arrays are regularly arranged in the treated area with a spacing of ~100 um (Figure 2c). It is worth noting that the height and the spacing of the microgroove arrays could be precisely controlled by adjusting the reasonable laser power, pulse number and scanning periods. In addition, due to the smaller thermal impact of the femtosecond laser in the processing area, the induced microgrooves have a high size accuracy, and their margin is smooth (Figure 2c). Moreover, some nanoparticles (200~500 nm) were induced on the microgrooves under the effect of debris deposition during the laser ablation process (Figure 2d), which is important for achieving the extreme wetting abilities of the prepared sample. In the present study, the laser-induced surface was superhydrophilic with a small contact angle, which is mainly related to the micro/nanostructures induced by the debris deposition between the laser and the materials.

### 3.2. The Mechanism of Bubble Directional Transmission on the Slippery Surface during the Fabrication of LIAMSS

**Figure 3:**
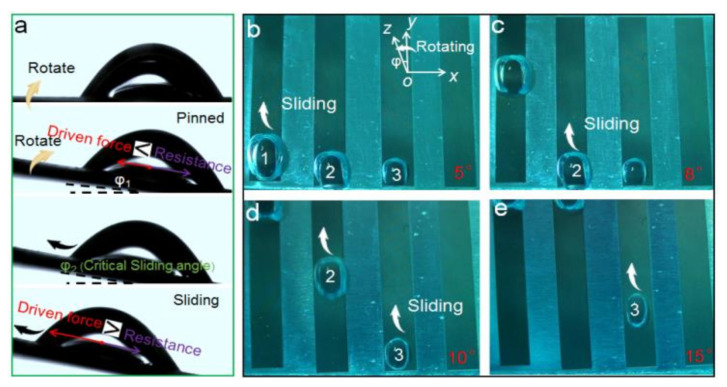
The directional transmission process of the underwater bubble on the restricted LIAMSS. (**a**) Side view of bubble sliding with the increase in incline angle. (**b**–**e**) The sliding process of underwater bubbles at different volumes on the restricted LIAMSS. It can be clearly seen that the bubbles with different volumes start to slide on the inclined LIAMSS at different rotating angles, which is mainly induced by the variation in bubble buoyancy along the incline direction. The adhesion force between the bubble and the oil layer is also important in controlling the directional movement of bubbles, which could be regarded as the viscous resistance during the sliding process.

The directional manipulation of underwater bubbles on the LIAMSS is vividly illustrated in Figure 3. It can be seen that when the bubble contacts the LIAMSS, it would adhere to the surface because of the adhesive force between the lubricant and bubble (Figure 3a). With the increase in incline angle (φ1), the bubble is driven along in the inclined direction induced by the buoyancy in the liquid, which is equal to the horizontal component of buoyancy along the incline direction of LIAMSS. It should be noted that the driving force is mainly related to the bubble volume and inclined angle; when the bubble volume is fixed, the driving force increases with the increase in incline angle. When the driving force induced by the buoyancy is greater than the resistance induced by the contact angle hysteresis, the bubble slides along the inclined LIAMSS, and the incline angle is defined as the critical sliding angle (φ2). It is worth noting that the critical sliding angle is closely related to the bubble volume (Figure 3b–e). It can be seen that three bubbles with different volumes are deposited on the LIAMSS. With the increase in rotating angle, the biggest bubble (marked 1) slides along the surface first because of the small driving force required, and the smallest bubble (marked 3) starts to move at the highest rotating angle (15°).

**Figure 4:**
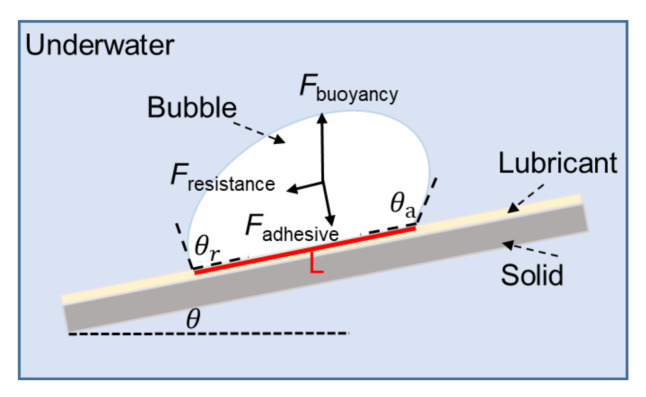
The mechanical analysis of the underwater bubble when it slips along the inclined LIAMSS. It is shown that the mechanism of underwater bubble sliding on the inclined slippery surface is related to the competition between the driving force induced by the underwater buoyancy and the resistance force induced by the contact angle hysteresis.

In order to study the mechanism of bubble directional transmission on the slippery surface, a mechanical analysis of the bubble sliding along the inclined LIAMSS is proposed by constructing a theoretical model, as in Figure 4. The main driving force of bubble sliding can be calculated by the following equation:

F_driven_ = F_buoyancy_·sinθ = ρ·g·V·sinθ (1)

where ρ, g and V denote the water density, gravitational acceleration, and the bubble volume, respectively. It can be seen that the driving force here is mainly induced by bubble buoyancy, which is equal to the horizontal component of buoyancy along the inclined direction of SLIPS. It is mainly related to the bubble volume and incline angle. When the bubble volume is fixed, the driving force increases with the increase in incline angle.

In addition, according to previous studies, the resistance can be divided into two parts: one is the contact angle hysteresis (CAH) resistance induced by the adhesion force between the bubble and the lubricant, and the other is the drag force during the sliding process [16]. The resistance of CAH can be expressed as follows [35]:

F_CAH_ = γ·L·(cosθ_r_ − cosθ_a_) (2)

where γ is the surface tension of water, L is the line length along the axis between the bubble and the surface, and θ_r_ and θ_a_ represent the advancing and receding angles of the bubble, respectively. In principle, the F_CAH_ determines whether the bubble starts to move on the surface. Therefore, with the increase in tilted angle, the F_CAH_ would increase with the increase in contact angle hysteresis. When the bubble starts to move at a certain tilted angle, the F_CAH_ stays the same.

**Figure 5:**
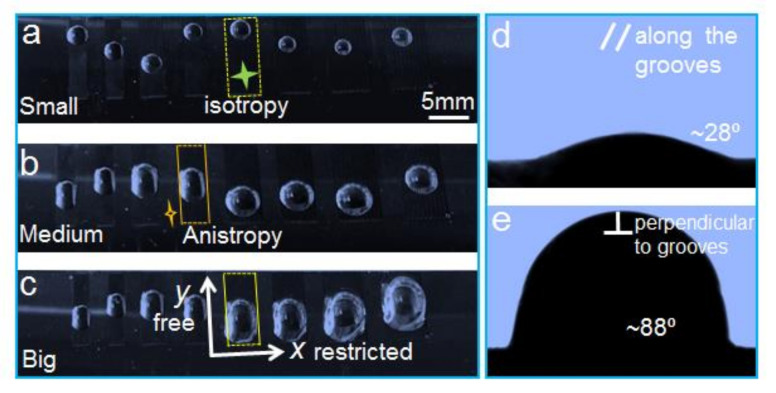
The anisotropic spreading of underwater bubbles on the restricted LIAMSS. (**a**–**c**) With the increase in bubble volume, the anisotropy spreading behavior becomes more and more obvious. (**d**) The bubble contact angle along the grooves. (**e**) The bubble contact angle perpendicular to the grooves. It is clearly seen that the BCA perpendicular to the grooves is much larger than that along the grooves, which is mainly induced by the different energy barrier in the two directions. Due to the restriction of the fabrication area, the bubble contact angle would become larger when the bubble volume increases.

Additionally, we find that the bubble’s sliding behavior on the LIAMSS has obvious anisotropy (Figure 5), which contributes to the difference in contact angles along and perpendicular to the grooves. It can be seen that when the bubble volume is small (Figure 5a), the bubble spreads along the groove direction without any restriction. With the increase in bubble volume, some bubbles are restricted in the perpendicular direction of the grooves (Figure 5b,c), so the contact angles along and perpendicular to the grooves have the significant difference of 60° (Figure 5d,e). It is worth noting that the difference between the contact angles along and perpendicular to the grooves is influenced by the bubble volume and the restricted area.

**Figure 6:**
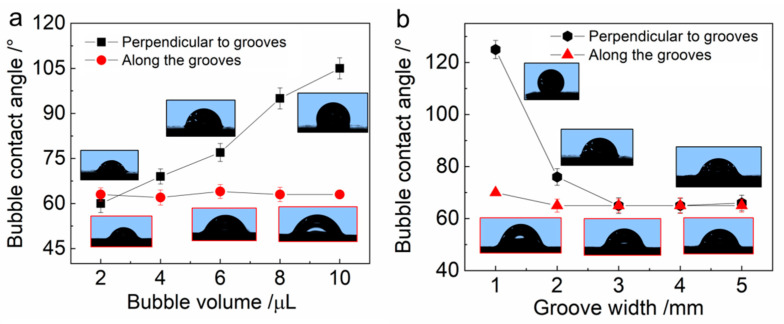
The influence of bubble volume (**a**) and groove width (**b**) on the bubble contact angle along the grooves and perpendicular to the grooves. It can be seen that the bubble volume and groove width have a significant effect on the bubble contact angle perpendicular to the grooves, which is mainly induced by the restriction of the slippery area during the bubble’s anisotropic spreading.

In order to explore the influence of bubble volume and slippery area width on bubble anisotropic spreading, we performed some quantitative experiments to measure the variation in bubble contact angle (BCA) under different bubble volumes and groove widths (Figure 6). It can be seen that the bubble volume has a great influence on the variation in BCA perpendicular to the grooves with the same groove width (2 mm), while the BCA along the grooves shows no obvious change (Figure 6a). With the increase in bubble volume, the bubble begins anisotropic spreading on the restricted LIAMSS, such that the BCA perpendicular to the grooves would increase with the increase in bubble volume. A similar phenomenon also occurs in the variation of BCA with a different groove width at the same bubble volume (6 μL, Figure 6b). When the bubble spreads on the LIAMSS with a smaller groove width, the bubble shows a stronger anisotropy because of the restriction perpendicular to the grooves, such that the BCA perpendicular to the grooves is much larger than that along the grooves. With the increase in groove width, the anisotropic spreading of the bubble becomes weaker and weaker, and so the BCAs in both directions show almost no obvious difference.

## 4. Conclusions

In summary, inspired by the unique lubricant features of pitcher plants, we have fabricated a kind of liquid-infused microgrooved slippery surface on the basis of femtosecond laser scanning and a lubrication process. Compared with traditional superhydrophobic/superhydrophilic surfaces, SLIPS possesses excellent repellency to various liquids with different surface tensions, and could remain effective after long-term immersion in liquid medium for continuous bubble manipulation processes. On the basis of this LIAMSS, we manipulated underwater bubbles in a directional path, and we also found that the bubbles show an anisotropic spreading behavior when on it. The primary underlying mechanism of bubble directional manipulation is the competition between the buoyancy and the resistance, including CAH and drag resistance. Additionally, the bubble shows obvious anisotropy on the LIAMSS with the increase in volume because of the restriction of the slippery area, and the bubble contact angle perpendicular to the grooved region is about 88° when the bubble volume is 5 μL. Finally, we explored the influence of bubble volume and groove width on the anisotropic spreading of bubbles on the restricted LIAMSS by measuring the variation in BCA along and perpendicular to the grooves. We believe the present study of bubble directional transmission behavior could serve as part of a gas collection system, with broad potential applications.
